# Sex-Related Differences in Immune Response and Symptomatic Manifestations to Infection with *Leishmania* Species

**DOI:** 10.1155/2019/4103819

**Published:** 2019-01-10

**Authors:** Ryan D. Lockard, Mary E. Wilson, Nilda E. Rodríguez

**Affiliations:** ^1^Department of Biology, University of Northern Iowa, Cedar Falls, IA 50614, USA; ^2^Departments of Internal Medicine and Microbiology, University of Iowa, Iowa City, IA 52242, USA; ^3^Veterans Affairs Medical Center, Iowa City, IA 52240, USA

## Abstract

Worldwide, an estimated 12 million people are infected with *Leishmania* spp. and an additional 350 million are at risk of infection. Leishmania are intracellular parasites that cause disease by suppressing macrophage microbicidal responses. Infection can remain asymptomatic or lead to a spectrum of diseases including cutaneous, mucocutaneous, and visceral leishmaniasis. Ultimately, the combination of both pathogen and host factors determines the outcome of infection. Leishmaniasis, as well as numerous other infectious diseases, exhibits sex-related differences that cannot be explained solely in terms of environmental exposure or healthcare access. Furthermore, transcriptomic evidence is revealing that biological sex is a variable impacting physiology, immune response, drug metabolism, and consequently, the progression of disease. Herein, we review the distribution, morbidity, and mortality among male and female leishmaniasis patients. Additionally, we discuss experimental findings and new avenues of research concerning sex-specific responses in cutaneous and visceral leishmaniasis. The limitations of current therapies and the emergence of drug-resistant parasites underscore the need for new treatments that could harness the host immune response. As such, understanding the mechanisms driving the differential immune response and disease outcome of males versus females is a necessary step in the development of safer and more effective treatments against leishmaniasis.

## 1. Introduction

### 1.1. Leishmaniasis

*Leishmania* are parasitic protozoa endemic in 98 tropical and subtropical countries. Worldwide, 12 million people are infected with *Leishmania* spp. and an additional 350 million are at risk of infection [1, 2]. *Leishmania* spp. are intracellular parasites that cause a spectrum of human diseases called leishmaniasis, including cutaneous (CL), mucocutaneous (ML), and visceral leishmaniasis (VL) as prominent forms [1, 3]. Post-kala-azar dermal leishmaniasis (PKDL) is a complication of VL, and there are several additional disseminated forms of cutaneous disease [1, 2]. *Leishmania* spp. life stages include the promastigote form, which converts to the infective metacyclic form in the gut of a phlebotomine sand fly vector, and the obligate intracellular amastigote present in the phagocytes of a mammalian host. *Leishmania* spp. have a number of mechanisms to subvert the microbicidal activity of the host macrophages [4]. Progressive leishmaniasis is characterized by replication of amastigotes and the spread of parasites to additional macrophages, leading to localized or disseminated disease manifestations [3].

VL is the most severe clinical form of leishmaniasis, and symptomatic VL is often fatal if left untreated. Up to 500,000 new cases of VL are estimated on a yearly basis, along with about 50,000 reported deaths [2]. Treatment modalities for VL are complicated by high cost, toxicity, and the need for lengthy and often parenteral administration. An increasing concern is the emergence of drug-resistant Leishmania parasites in some endemic areas, particularly among *L. donovani* strains in India [2, 5, 6]. These limitations highlight the importance of developing new approaches to therapy. In particular, approaches that synergize with the host immune response as well as natural determinants of susceptibility provide potential targets to interfere with disease progression.

### 1.2. Biological Sex as a Variable in Physiology and Disease

Large-scale transcriptomic studies are shedding light on the various ways that biological sex impacts physiology and as a consequence, disease outcomes. Males and females have a similar number of genes, differing only in those encoded by the sex chromosomes. However, in various tissues, the expression of many transcripts can differ significantly between the sexes. In humans, this includes the brain, heart, liver, and peripheral blood mononuclear cells. Furthermore, the number of biased transcripts and their respective fold difference varies from tissue to tissue between both sexes [7–11].

Sex-related differences in immunophysiology may underlie distinct male or female susceptibility to certain cancers and autoimmune conditions as well as infectious diseases, including several that are caused by parasites [8, 12–16]. Both pathogen and host factors likely drive these dichotomous rates in disease [16–18]. It cannot be excluded that social dynamics and gender-associated behavior might play roles in the disposition to seek medical care and consequently, disease outcome [13, 14, 16, 17]. However, often these sex differences cannot be explained by environmental exposure or healthcare access, suggesting a physiological basis [13, 14, 16–20]. Biological differences between the sexes have been shown in animal models and in a number of human disease states. Furthermore, sex-associated differences have been observed in cells of male versus female origin, strongly supporting a role for biological sex in disease development [7, 9–12, 16–19].

In this review, we will explore epidemiological patterns of sex-related differences in cutaneous and visceral leishmaniasis, in addition to other manifestations of symptomatic infection. We also outline ongoing efforts to elucidate the immune mechanisms contributing to sex-specific responses in disease.

## 2. Cutaneous Leishmaniasis

### 2.1. Epidemiological Evidence of Sex Bias in Old World CL

Old World CL is caused primarily by *L. aethiopica*, *L. major*, and *L. tropica*, while in the New World, *L. mexicana* and species of the *Leishmania* (*Viannia*) (*V.*) subgenus are largely responsible for causing CL [21, 22]. Differential incidence of CL between the sexes has been reported in multiple endemic regions of the Old World. Whether CL is more common in males versus females appears to differ depending on multiple variables. It is likely that a complex interaction between environmental, host gender and biological factors, and the infective *Leishmania* species, determines whether there is a sex bias and, if so, the direction of the bias [23–29].

### 2.2. Sex-Dependent Differences in Experimental Models of Old World CL

During infection with the Old World species *L. major*, the resistant and susceptible phenotypes of C57BL/6 and BALB/c murine models are determined by their respective Th1- and Th2-mediated CD4+ T cell responses [30, 31]. Sex biases have been documented in murine *L. major* infection; however, whether it is males or females that develop more severe disease is highly dependent on the model. This suggests that sex-dependent responses are driven by factors that are differentially expressed in male versus female mice and their distinct interactions with parasites [32–34].

Studies using wild-type (WT) C57BL/6 mice showed that a low-dose *L. major* inoculum, which resembles natural infection, resulted in higher parasite burdens and parasite spread to the spleens of female than male mice. In addition, cells from draining lymph nodes showed that females have a cytokine profile resembling Th2 responses, with higher interleukin (IL)-4 and lower interferon-*γ* (IFN-*γ*) than male mice. Interestingly, in this study, neither macrophages, dendritic cells, nor lymphocytes were involved in driving the biased cytokine profile and disease outcome. The authors hypothesized that factors present at the time of inoculation or shortly thereafter might be involved in driving the higher susceptibility of females. These could include stromal tissue, fibroblasts, keratinocytes, and/or hormones [31]. Several other lines of evidence support a role for keratinocytes and hormones in mediating sex-specific differences at the inoculation site. First, many skin diseases show a sex bias in prevalence, severity, and mortality [35, 36]. Second, sex hormones are known to mediate differences in skin structure and physiology between males and females [35, 36]. Third, keratinocytes and sebaceous glands can synthesize sex hormones [36, 37]. In particular, keratinocytes can synthesize sex steroids de novo, which in turn can exert autocrine and paracrine effects in various targets, including immune cells, potentially contributing to sex-related immune responses [37]. Furthermore, studies in other systems have shown that IL-6, a cytokine produced by keratinocytes and stromal cells, among others, can skew T cell responses away from Th1- and towards Th2-type responses [38, 39]. Overall, these studies suggest complex host-pathogen interactions at the inoculation site, although further studies will be necessary to dissect the factors contributing to sex-biased responses at the earliest stages of infection.

Additional factors with the potential to drive sex-dependent responses during or shortly after Leishmania inoculation include tissue-specific differences in resident immune cell populations. For example, in C57BL/6 mice, males have more resident neutrophils and nonclassical monocytes in the spleens than their female counterparts, whereas there are twice as many macrophages, B cells, and CD4+ T cells in the pleural and peritoneal cavities of females than males [40, 41]. However, no sex differences were observed in the bone marrow [40]. It is possible that variations in cellular composition might be driven by tissue-specific chemokines [41].

Sex-dependent differences have also been shown in immune cell infiltration. For example, a model of acute inflammation showed higher neutrophil and monocyte recruitment in male than female C57BL/6 mice [40]. Moreover, sex-dependent responses have been observed in eosinophil infiltration during *L. major* infection, although the role of eosinophils in leishmaniasis is complex [33, 42]. In vitro studies suggested that eosinophils are microbicidal toward *L. major* and *L. amazonensis* and increased numbers of eosinophils in vivo correlate with resistance to murine *L. mexicana* and *L. infantum* infection [42]. In contrast, Slapnickova et al. found a significant correlation between eosinophil infiltration and increased parasite loads in male mice but not female mice infected with *L. major*. This suggests a male-specific detrimental role for eosinophils [33].

Murine host polymorphic genes associated with eosinophil infiltration into lymph nodes mapped to four autosomal loci, two of which showed a cooperative effect (*Lmr15*, chr 11; *Lmr26*; chr 9) and two of which apparently functioned independently (*Lmr14*, chr 2; *Lmr25*, chr 5). Interestingly, *Lmr14* was associated with eosinophil infiltration in male mice but not female mice. The positive correlation between parasite load and higher eosinophil infiltration in males was suggested to reflect the chronic inflammatory state in *L. major* infection [33]. These results form part of a growing number of studies showing the potential involvement of autosomal genes in controlling pathogen tropism, immune responses, and the sex-dependent outcome of infection [13, 16, 33]. In summary, it can be speculated that at the inoculation site, qualitative and quantitative differences in cellular composition and inflammatory signals will affect the development and maintenance of the immune response and the outcome of infection between males and females [2, 33, 42].

Sex-based differences have also been described in patients and in animal models of infection with *L. tropica*, another Old World species. In addition to cutaneous lesions, *L. tropica* infection can result in VL, simultaneous VL and disseminated CL, or viscerotropic disease, a hybrid of cutaneous and visceral symptoms that is defined by the systemic spread of parasites [32, 43–45]. Epidemiological data suggest that different clinical syndromes due to *L. tropica* infection vary also in their sex bias. CL, the most common presentation of *L. tropica* infection, seems to be more prevalent among females in some endemic areas, whereas males are more likely to develop viscerotropic leishmaniasis [26, 28, 44]. However, the mechanisms responsible for the spectrum of disease manifestations and the variation in sex-dependent outcomes have been elusive, due in part to the lack of an apt animal model [32].

Recently, Kobets et al. developed a suitable model to study the intersection between genotype, sex-dependent differences, and *Leishmania* species-specific responses. They used nine recombinant congenic mouse strains from STS/A (CcS/Dem) on a BALB/c background to examine the progress of *L. major* and *L. tropica* infection [32]. Compared to *L. tropica*, infection with *L. major* progressed faster and showed increased pathology in all but one of the strains of mice. In some, but not all strains, males developed larger skin lesions than females. However, during infection with *L. tropica*, females of most strains formed larger skin lesions. This did not correlate with higher parasite burdens in draining lymph nodes of females, suggesting the involvement of other host factors, such as inflammatory mediators, in the sex-associated differences in skin lesions. For example, the greatest skin pathology to *L. tropica* infection was shown by females of the strain CcS-16, which also showed the highest expression of the C-C motif chemokine ligands (CCL) CCL3 and CCL5. These chemokines have been associated with resistance to *L. major*, although CCL3 has been related with chronic progressive *L. mexicana* disease [32]. The specific effects of CCL3 and CCL5 in *L. tropica* infection merit further study.

The complex interactions between genotype, parasite species, and sex-based disease manifestations are well illustrated in the CcS-11 mouse strain. This strain showed only moderate susceptibility to *L. major*-induced lesions but succumbed to *L. tropica* infection. By week 14, female mice developed small lesions that healed into nodules; however, by weeks 32-42, about half the animals had died. Males were more resistant to skin pathology as they developed only small or no nodules, yet most male mice died earlier, by week 18. Histopathological examination showed no parasite dissemination into the spleen of CcS-11 mice. In contrast, *L. tropica* visceralizes to the spleens of BALB/c mice and several other recombinant strains used in this study [32, 43]. Together, these results suggest that the differential response to infection between males and females, if present, includes a combination of factors such as host genetic background, organ-specific responses, and the *Leishmania* species.

### 2.3. Epidemiological Evidence of Sex Bias in New World CL

Unlike Old World CL, data from the New World document a higher incidence of CL in males than females in Mexico and Colombia [46–48]. Similar reports from across Brazil show that males develop cutaneous manifestations of leishmaniasis at higher rates than females [49–53]. A nationwide study found increased CL in males versus females under age 1. The predominance in male infants could be driven by minipuberty, a transient postnatal increase in sex steroid levels that shows clear hormonal differences between boys and girls and has been implicated in other sex-biased infectious diseases [54, 55]. Overall, the prevalence of CL among males increased at puberty, reached its highest level in adulthood, and decreased in the elderly population. This occurred despite the fact that males likely do not experience increased parasite exposure, suggesting that the observed sex dichotomy has a biological basis [54, 56]. Together, these studies support the hypothesis that inherent biological factors, perhaps together with gender-related behavior, place males at higher risk of New World CL.

### 2.4. Sex-Dependent Differences in Experimental Models of New World CL

#### 2.4.1. *L. mexicana*

Mice lacking the IL-4 receptor*α* (R*α*) are resistant and do not form lesions during *L. mexicana* infection. Since IL-4R*α* is a common subunit in the IL-4 and IL-13 receptors, these results suggest that either IL-4, IL-13, or both are involved in susceptibility [30]. To identify the critical cell populations, Bryson et al. studied infection in global- and CD4+ T cell-specific IL-4R*α*-deficient mice in a BALB/c background and compared these results to that in WT mice. These data show that IL-4R*α* from CD4+ T cells, but not from neutrophils and macrophages, is implicated in disease progression by facilitating Th2 responses. Strikingly, the splenocyte cytokine response differed between CD4+ T cell-specific IL-4R*α*-deficient male and female mice. Splenocytes from males expressed higher levels of IL-4, IL-5, IL-10, and immunoglobulin G1 (IgG1). This occurred despite the fact that mice of both sexes curtailed disease progression. However, while females healed the initial small lesions, sex-specific, IL-4R*α*-independent production of IL-4 led to lesion persistence in males [30].

In humans, *L. mexicana* infection can result in localized CL (LCL) with ulcers at the site of parasite inoculation or anergic diffuse cutaneous leishmaniasis (ADCL). ADCL is characterized by high parasite loads, suppressed cutaneous delayed-type hypersensitivity, and disfiguring nodules throughout the body. Innate immunity has been implicated in parasite dissemination in ADCL [3, 45]. Natural killer (NK) cells, components of innate immunity, have the potential to activate innate and adaptive immune responses and thus could play a role in the differential manifestations of LCL versus ADCL. Peripheral blood and tissue samples showed higher numbers of NK cells in LCL than ADCL patients. In addition, LCL patients showed increased expression of IFN-*γ* and tumor necrosis factor (TNF) in nonstimulated and lipophosphoglycan (LPG) stimulated NK cells, with greater levels in females than males [45]. Increased NK cell numbers and their associated production of IFN-*γ* and TNF facilitate macrophage activation and parasite control and might contribute to the increased resistance of females to *L. mexicana* infection [34, 45].

The aforementioned studies showed how differences in the immune response contribute to *L. mexicana* susceptibility among males. Although some sex-specific immune responses may be determined by the male or female genotype, some are modulated by circulating sex hormones [13, 16]. Several studies discussed herein have examined sex steroids as a variable in infection using animal models of leishmaniasis [34, 57–59]. Additionally, a role for sex steroids in disease progression is implied by the epidemiological data [54, 60–62].

In addition to modulating host susceptibility, sex hormones could directly affect pathogens [16, 19, 63, 64]. Treatment of *L. mexicana* promastigotes with physiological doses of dihydrotestosterone (DHT), the main circulating androgen in males, increased parasite growth [13, 64]. In addition, DHT-treated parasites increased the macrophage infection rate and parasite load and were more resistant to the macrophage microbicidal attack. Furthermore, male BALB/c mice infected with DHT-treated parasites developed larger lesions and contained more parasites per area than those infected with nontreated parasites [64]. Overall, these results suggest that interactions between sex steroids, parasite virulence, immune responses, and host susceptibility determine the final outcome of Leishmania infection.

#### 2.4.2. Leishmania (Viannia) Species

Sex differences in the severity of experimental leishmaniasis are not limited to species of the *L. Leishmania* subgenus (*L. major*, *L. mexicana*). Studies in hamster models of cutaneous infection with either *L.* (*V.*) *guyanensis* or *L.* (*V.*) *panamensis* led to higher parasite loads in males than females. Although overall infection severity was higher with *L.* (*V.*) *guyanensis*, the sex dichotomy was most pronounced in *L.* (*V.*) *panamensis*-infected animals [59].

A potential role for sex steroids in driving the differential responses to *L.* (*V.*) *panamensis* was investigated in studies of estrogen-treated male or testosterone-treated female hamsters. There was an increase in the size of lesions of hamsters treated with testosterone, whereas estrogen treatment had no discernible effects. This was consistent with the observation that the augmented size and necrosis of lesions due to *L.* (*V.*) *panamensis* developed only in adult male hamsters that have reached biological maturity. These sex differences correlated with the abundance of skin lesion transcripts encoding IL-4, IL-10, and transforming growth factor-*β* (TGF-*β*), but not IFN-*γ* or IL-12 [59]. *L.* (*V.*) *guyanensis* infection resulted in a contrasting pattern, in that adult males developed an increase in necrosis but not in lesion size compared to juvenile males. Furthermore, adult female hamsters had smaller lesions than juveniles infected with *L.* (*V.*) *guyanensis*, consistent with a protective role for estrogen [59]. Similar sex-based differences have been observed in other infectious disease models including *Staphylococcus aureus*, in which female resistance to dermonecrosis is associated with estrogen and its downstream effects [35].

## 3. Visceral Leishmaniasis

### 3.1. Epidemiological Evidence of Sex Bias in Old World VL

VL, known in some endemic areas as kala-azar, is a systemic disease characterized by enlargement of the spleen and liver as well as progressive immunosuppression [2, 65]. VL is caused by *L. infantum* in the New World and either *L. infantum* or *L. donovani* in the Old World [2, 21, 65]. Considering VL patients from Brazil, East Africa, India, and Nepal, Harhay and colleagues concluded that despite variations in the ratios of males-to-females, males accounted for more cases of VL across all regions. These observations could result from parasite, host, societal factors, or some combination of these variables [5]. An illustration is VL caused by *L. donovani* in the Indian subcontinent and other areas, where gender and biological factors could contribute to variations in disease severity between the sexes [34, 61, 66–72]. Reports from India are uncertain regarding a sex bias in PKDL, a cutaneous complication sometimes observed after VL treatment; whereas some studies indicate no difference, others show a greater incidence of PKDL in males [69, 73, 74]. In India, conclusions about the involvement of biological factors are complicated by the likely underreported numbers of adult females with symptomatic VL, which may result from gender disparities in healthcare access in the region [61]. However, in some endemic areas (e.g., Northeast Brazil), studies provide evidence that the male predominance in VL prevails even in situations of similar infection rates between the sexes, as discussed herein [60, 75].

Sex-associated differences in antileishmanial IgG titers have also been reported, although not always in the same direction. Among VL patients from India, males had higher IgG titers whereas Sudanese females with VL had higher serologic titers than males. In both populations, sex differences were observed in subjects aged 16 or older, raising the possible involvement of sex hormones [76]. Although the physiologic consequence of serologic differences is not known, elevated leishmania-specific IgG has been identified as a marker of subjects who will progress from asymptomatic to symptomatic VL [77].

### 3.2. Sex-Dependent Differences in Experimental Models of Old World VL

Several studies have set out to investigate the mechanisms underlying the sex bias observed in Old World VL epidemiological data. Compared to females, male hamsters infected with *L. donovani* exhibited higher parasite loads. Furthermore, in both sexes, estradiol lowered whereas testosterone increased infection [78]. In murine macrophages, testosterone has been shown to promote *L. donovani* uptake and parasite load, and decrease p38 mitogen-activated protein kinase (MAPK) activation, which may lead to a lower antimicrobial response and promote parasite survival [79–82]. In contrast, exposure to 17*β*-estradiol has no impact on parasite load [81]. Overall, these results implicate sex hormones in the modulation of *L. donovani* infection. The potentially detrimental effects of testosterone would likely hold more significance in the infection of males than females.

### 3.3. Epidemiological Evidence of Sex Bias in New World VL

In Brazil, where *L. infantum* is endemic, the epidemiological data have largely shown a male predominance in VL [54, 83–88]. In addition, one nationwide study found higher levels of discontinued therapy among male VL patients. It is unknown whether gender-related behavior explains higher rates of treatment cessation among men or if sex-related biological differences lead to more adverse effects, and thus, higher treatment withdrawal [86]. In the same study, males (*n* = 1,168) represented a greater number of deaths caused by VL compared to females (*n* = 611), similar to other reports from Brazil as well as in Southern Iraq where *L. donovani* is endemic [84–86, 89, 90]. Furthermore, morbidity and mortality associated with HIV and *Leishmania* coinfection in Brazil and Spain are reported to be higher in males [84, 85, 87, 89, 91]. However, epidemiological studies cannot always discern whether differences between males and females are due to gender-related or biological factors.

Data from the state of Rio Grande do Norte, Brazil, have consistently demonstrated a male bias in VL [60, 62, 92]. *Lutzomyia longipalpis*, the sand fly vector of *L. infantum*, is peridomestic, and males and females have similar rates of infection [60, 62, 93]. However, after the age of ten, males were more likely to develop symptomatic disease than females. This age range approximately corresponds with the start of puberty [60, 62]. An expanded analysis of 1,967 cases of VL reported from across the state showed the same pattern of male bias in disease after puberty. Likewise, a nonsignificant male bias among VL subjects under the age of 1 may be related to minipuberty, which has been implicated in other studies of leishmaniasis [54, 62]. Overall, males accounted for 66% of symptomatic cases [62]. Other studies in Brazil have found comparable rates of infection between the sexes but did not always differentiate between early childhood and postpubertal patients [54, 94–96]. Those studies could be complicated by the fact that VL has been a disease predominant in children under the age of 10, although this epidemiological pattern is now changing [97]. The increased frequency of disease in postpubertal males could result from a combination of factors, including biological variables such as the anti-inflammatory properties of testosterone and sex-based genetic differences, possibly in combination with gender-related behavioral patterns [54, 62, 86, 87, 98, 99].

### 3.4. Sex-Dependent Differences in Experimental Models of New World VL

Collectively, epidemiological studies of New World VL support the concept that the interaction between gender-based behavior and biological sex could determine the outcome of disease. Experimental studies are necessary to elucidate the biological aspects of sex-dependent responses to infection with visceralizing species of *Leishmania*.

### 3.4.1. Sex-Dependent Differences at the Macrophage Level: Receptors, Parasite Load, and Potential Implications for Leishmaniasis

*(1) Macrophage Receptors*. Phagocytic and antimicrobial differences in macrophages of male versus female origin have been shown in various infectious diseases including *Cryptococcus neoformans*, group B streptococci, and *Paracoccidioides brasiliensis* [19, 20, 41]. Recently, our group examined infection levels in C57BL/6 macrophages of male and female origin. In the initial hour of infection with LcJ, a clonal line of a Brazilian *L. infantum* strain, macrophages from male mice showed higher percent of infection and parasite load. Leishmania uptake is mediated by various receptors including mannose, fibronectin, Fc*γ* receptor, complement receptor-1 (CR1), and CR3 [100–104]. The observation of sex-biased uptake of LcJ promastigotes suggests receptor differences in C57BL/6 macrophages of male versus female origin [62]. Recognition by macrophage receptors is among the earliest interactions between Leishmania and its host, and parasite survival is impacted by differential uptake [100, 104]. As such, sex-specific differences at this stage could have a major impact on infection outcome.

Transcriptomic data have shown differential expression of surface and downstream signaling molecules in macrophages of male versus female origin. These differences are far more extensive than those originating only from the sex chromosomes. Many variations arise from dissimilar regulation in epigenetics and gene expression [7, 10, 105]. For example, mouse macrophages from males and females showed over 30% of genes differentially expressed. Additionally, transregulation of expression quantitative trait loci was mostly sex specific. The differential gene expression was independent of exogenously added sex steroids, as these were ex vivo experiments [105]. However, it cannot be ruled out that endogenous sex hormones could have induced long-lasting epigenetic changes nor can it be excluded that there were intracrine or autocrine effects of sex hormones released by macrophages in vitro [37]. Overall, these data showed a strong effect of biological sex in both gene expression and transregulation, thus, supporting the rationale of segregating data analysis by sex [37, 105].

Likewise, macrophages from male and female donors differentially express various transcripts including CD14, cell surface adhesion proteins, signaling molecules, transcription factors, and Toll-like receptors (TLRs) [7, 10, 12, 13, 41, 106]. To facilitate their survival, *Leishmania* spp. interact with TLR-2, TLR-9, and TLR/IL-1R signaling cascades [107, 108]. Similarly, *L. infantum* increases the transcripts of phagocytosis receptors implicated in parasite survival while decreasing those associated with parasite clearance [109]. Differential use of macrophage receptors leads to distinct entry and parasite survival [100, 103, 104]. As such, future studies should examine whether sex differences in the types or abundance of receptors involved in parasite recognition and entry contribute to the differential responses of male- versus female-derived macrophages.

*(2) Parasite Load*. As we previously showed that LcJ and WT *L. infantum* promastigotes could utilize different entry pathways, the sex-biased uptake of attenuated parasites led us to examine the kinetics of infection using a WT strain of *L. infantum* from Brazil [62, 100]. Uptake and parasite load of WT promastigotes were similar between C57BL/6 macrophages of male and female origin in the initial 24 hours. In contrast, by 72 hours, parasite loads were higher in macrophages from males. Thus, despite similar uptake of WT parasites, macrophages from females contained the infection better [62].

While more comprehensive studies are necessary to determine the mechanisms underlying these sex-dependent differences, the 72-hour kinetics implicate the involvement of nitric oxide (NO) [62, 110]. Related studies with *L. mexicana* showed increased NO production by female-derived DBA/2 macrophages leading to better control of infection [34, 58]. Furthermore, treatment with estrogen increased NO and microbicidal activity in *L. mexicana*-infected DBA/2 macrophages from both sexes [34, 58]. Sex-dependent responses in *L. mexicana* infection could depend on the host genotype, as studies with C57BL/6 macrophages did not show the same results [57, 58]. Thus, sex-dependent antimicrobial responses are determined by the combination of the host genotype, sex hormones, and *Leishmania* species [34, 57, 58]. Collectively, these results indicate that inherent differences at the macrophage level, which may be further modulated by sex hormones, could be factors driving the sex dichotomy in disease.

### 3.4.2. Sex-Dependent Differences in Granuloma Formation: Potential Implications for VL

To further explore the mechanisms mediating sex differences, we examined liver parasite loads in *L. infantum*-infected BALB/c and C57BL/6 mice. In both strains, male mice had higher parasite burdens than females, suggesting that across genetic backgrounds, males are more susceptible to *L. infantum* infection [62]. In the mouse model, containment of parasite growth in the liver correlates with the induction of Th1-mediated immune responses, which interact with TLR7 in the formation of liver granulomas [2, 110–112].

Immune cells from females have higher expression of TLR7, a gene present in the X chromosome. In humans and mice, females avoid doubling the expression of genes encoded on the X chromosome by inactivating the second copy [7, 13, 113, 114]. However, this is not a comprehensive shutdown and at least 15% of the genes in the second X chromosome escape inactivation, either partially or completely [7, 10, 12, 13, 113]. The X chromosome contains a high number of immune-related genes, and incomplete gene inactivation could facilitate some amplified immune responses in females [9, 12]. Escaping gene inactivation might not be random, and it has been proposed that some genes, such as TLR7, are biased to be overexpressed in females [7, 9, 13]. Thus, it can be speculated that female tissues will respond to infections with stronger granuloma formation. In agreement with this notion, the BALB/c and C57BL/6 models of *Mycobacterium tuberculosis* infection have shown that lung granulomas form earlier and reach a larger size in female than in male mice [115, 116]. Furthermore, castration of male BALB/c mice improved granuloma formation and survival, implying an adverse effect of testosterone in granuloma development [115]. Taken together, these studies suggest that females could more readily initiate and maintain granulomatous immune responses. Whether granulomas contribute to the sex-biased responses observed in *L. infantum* infection deserves further investigation.

### 3.4.3. Sex-Dependent Differences in Cytokine Responses

In the mouse model of *L. major* infection, a distinct dichotomy of Th1- or Th2-mediated responses results in cure or disease, respectively. The immune responses implicated in experimental CL systems involving other *Leishmania* species as well as in VL are more complex. Similar to experimental models of CL, other than *L. major* BALB/c mice, a clear-cut Th2 response is absent in *L. infantum*-infected animals or VL patients. Nonetheless, Th1 responses have been associated with resolution of either asymptomatic or symptomatic infection in all models and infections examined [65, 117].

In response to anti-CD3 and anti-CD28 stimulation, naive T cells from female donors produce more IFN-*γ* and express higher levels of IL-12R*β*, as well as molecules associated with higher cytotoxic activity [14, 118]. Furthermore, animal infection models and vaccination studies in humans have shown that females have increased expression of several molecules involved in the TLR pathway and Th1 responses, including TLR8, myeloid differentiation primary response 88 (MyD88), and nuclear factor-kappaB (NF*κ*B) [13, 114]. Overall, T cells from females displayed a stronger proinflammatory profile, which became increasingly more so with repeated stimulation. In contrast, T cells from males displayed a mixed profile, with the inflammatory cytokine IL-17A, the eosinophil-recruiting cytokine IL-5, and the anti-inflammatory cytokine IL-10 [118]. Interestingly, about half of the genes overexpressed in T cells from females had estrogen-responsive elements (EREs), suggesting a regulatory role for sex steroids [13, 16, 114, 118]. However, the mechanisms underlying sex-biased T cell immune responses have not been elucidated.

Cytokine expression assays in male and female C57BL/6 mice infected with *L. infantum* showed no difference in the serum levels of IL-12, a type 1 cytokine [2, 62]. In contrast, males had higher levels of serum TNF and IL-10, two cytokines implicated with exacerbated VL [2, 62, 65, 119–121]. Examination of cytokine expression in splenocytes showed that females have higher levels of the proinflammatory cytokine IL-1*β*, and IFN-*γ*, a key type 1 cytokine important for protective or curative immune responses. No sex-dependent difference was observed in IL-4, consistent with the observation that type 2 responses do not drive VL susceptibility [62, 65, 117]. A sex-based difference was also observed in splenocyte-derived IL-6 and IL-17, with males showing higher expression [62]. Our results are in agreement with animal models of inflammation in which splenocytes cultured from females produced higher IL-1, whereas splenocytes from males released higher levels of TNF and IL-6 [122].

IL-6 is a pleiotropic cytokine produced by many cell types [12, 38, 39]. As a consequence, its role in leishmaniasis has not been fully delineated. However, IL-6 has been implicated in the modulation of macrophage and T cell responses. For example, IL-6 has the potential to drive macrophages away from M1 (classical) activation leading to lower microbicidal activity and inhibition of proinflammatory cytokines, which could facilitate parasite growth [39, 109]. In addition, IL-6 facilitates type 2 cellular immune responses whereas it suppresses type 1 immunity by interfering with IFN-*γ* signaling [38].

Peripheral blood mononuclear cells from male donors have higher basal levels of IL-6 than cells from females, and this difference further increases upon stimulation [123]. In experimental *L. donovani* infection, IL-6 decreases IFN-*γ* leading to increased liver parasite load [124]. Similarly, among *L. infantum*-infected VL patients, IL-6 correlates with disease severity and death risk, both of which show a male predominance [86, 125]. An association between elevated IL-6 levels and more severe disease in males has also been documented among hepatocarcinoma patients [12, 15, 63].

In concert with TGF-*β*, IL-6 facilitates the induction of Th17-type effector cells, the main producers of IL-17 [39, 117, 120, 126, 127]. There is a balance between the development of Th17 and the differentiation of regulatory T cells (Tregs) in humans, and IL-6 appears to be a pivotal regulator of this equilibrium. IL-6 has been reported to inhibit the Treg-associated transcription factor FOXP3, and in combination with TGF-*β*, it has been shown to downregulate FOXP3 protein levels. On the other hand, TGF-*β* promotes Treg differentiation, possibly in a dose-dependent manner [126, 127]. There is abundant TGF-*β* in the organs of BALB/c mice as well as bone marrow of VL patients infected with *L. infantum*, although recent studies have shown that progressive visceral leishmaniasis in humans and mouse models is associated with IL-10-producing CD4+ T cells [128–132]. In addition to the well-studied type 1 CD4+ T cells, the ultimate outcome of chronic VL is the sum of the effects of adaptive cell populations expanded in the host, including B cells, CD8+ T cells, and CD4+ T cells expressing IFN-*γ* and inhibitory IL-10, as well as Tregs and Th17 cells.

Studies of IL-17 in VL have shown mixed results, with some studies suggesting a protective role whereas others suggest that it is detrimental to the host [117, 120]. IL-17A, the most studied member of the IL-17 family, is implicated in the expansion and recruitment of neutrophils. However, the ultimate effect of neutrophils in *Leishmania* spp. infection varies according to the tissue infected, the timing of recruitment, and their persistence at the infection site [2, 120]. Further examination of the effects of IL-17A in VL showed lower parasite load in the spleen and liver of *L. donovani*-infected IL-17A^−/−^ C57BL/6 mice. The absence of IL-17A led to increased IFN-*γ* production by CD4+ T cells and lower neutrophil recruitment. These results suggest that IL-17A facilitates VL progression by impairing IFN-*γ* responses while supporting damaging inflammation [117].

Furthermore, studies from our group and others suggest a link between IL-17 and sex-specific responses. For example, male donors have higher numbers of Th17-type cells in peripheral blood than females. In addition, naive and stimulated T cells from males secrete more IL-17 than those from females [118, 133]. Similarly, male C57BL/6 mice infected with *L. infantum* released significantly higher levels of IL-17 than females. Additionally, IL-6 increased slightly in C57BL/6 males [62]. IL-6 induces the development of Th17-type cells, and consequently, can facilitate IL-17 production [120]. Thus, higher IL-6 and IL-17 expression may converge to influence pathways underlying sex-dependent responses.

## 4. Transcriptomic Evidence of Sex-Dependent Immune Responses: Potential Implications for Leishmaniasis

Accumulating transcriptomic evidence is increasing our knowledge of sex-based differences in immunity [7, 106]. In a comprehensive study, Piasecka et al. examined transcriptional profiles from 500 males and 500 females before and after stimulation of whole blood samples with various microbial challenges. Of the 560 immune-related genes examined, 509 (91%) showed significant sex differences in response to at least one stimulus. One hundred eighty-one (36%) of the sex-biased genes were differentially expressed only after stimulation. Thus, some sex-specific differences in the immunotranscriptome are present in basal conditions while others are inducible [106].

In a similar study, microarrays of mononuclear blood cells of over 5,200 healthy human subjects showed about 1,000 transcripts differentially expressed between the sexes [7]. Many female predominant transcripts were associated with the Kyoto encyclopedia of gene and genome pathways termed as cytokine stimulus, response to interferon 1, and lymphocyte differentiation, whereas male predominant transcripts were related to the pathway named lysosomes [7, 134]. Some of the transcriptional differences decreased in postmenopausal women or increased in women using hormone-based contraceptives, suggesting a role for sex steroids. However, the number and magnitude of the most significantly changed transcripts did not correspond with the hormone status, indicating that sex-based transcriptional differences are driven by multiple mechanisms [7, 9].

Differences between male and female transcriptomes are highlighted by a meta-analysis of 22 microarray studies involving 2,500 healthy subjects. Samples from 15 tissues showed over 3,000 transcripts differentially expressed. The major differences were found in the brain and the heart. The liver, a target organ in VL, also showed a significant number of sex-biased transcriptional differences. Global analysis of the transcripts overexpressed in one sex or the other revealed that about one-third contained hormone-responsive elements, including androgen response elements, estrogen response elements, or both. Some of the differences were due to genes on sex chromosomes. However, almost two-thirds of the dichotomous transcripts were found in autosomal chromosomes and did not contain sex steroid binding sites [10]. It has been proposed that these transcripts could be controlled by sex-specific epigenetic modifications [10, 11]. Data from VL epidemiological studies show a higher male bias at puberty, coinciding with the spike of sex hormones, and a moderate bias into older age; these observations could be associated with the cumulative effects of sex steroids and sex-biased epigenetics [54, 60–62].

Similarly, a study of sex-associated differences in liver cell transcriptomes from equal numbers of male and female subjects (*n* = 224) showed over 1,200 transcripts differentially expressed between the sexes. Transcripts involved in lipid and drug metabolism showed the highest magnitude differences between males and females [11]. Given the expanding literature on the association between dyslipidemia and inflammatory states and the recent recognition of dyslipidemia in animals and humans with VL, these differences deserve exploration in the context of VL [135–137]. Furthermore, other studies have shown extensive sex-dependent differences in the expression of genes encoding enzymes involved in liver metabolic pathways, including the drug-metabolizing enzymes cytochrome P450s (CYPs) [8, 15, 138]. Differential expression of various CYPs results in distinct pharmacokinetics and pharmacodynamics between males and females [138]. Overall, vast transcriptome differences in the liver, an organ involved in VL pathogenesis as well as drug and lipid metabolism, emphasize the importance of tracking experimental and clinical differences in male versus female subjects.

## 5. Sex-Related Differences in the Outcome of Antileishmanial Treatment

Higher rates of treatment failure or more adverse effects among males have been shown in several studies. For example, in Colombia, male CL patients were more likely to maintain measurable parasite burdens following treatment with either miltefosine or meglumine antimoniate, although these results were not statistically significant [139]. In a related study of 318 CL patients in Belo Horizonte, Brazil, three times more males (*n* = 24) than females (*n* = 8) experienced relapse following treatment with meglumine antimoniate. However, in a hazard risk analysis, sex was not significantly associated with relapse [140].

In studies from the Indian subcontinent, male patients were at a greater risk of VL relapse after treatment with either miltefosine or liposomal amphotericin B [6, 141]. The differential responses between males and females could be due to male-biased healthcare access [141]. Sex differences in relapse rates might also result from biological factors such as differential drug metabolism and variations in immune responses [6]. In Nepal and Bihar, India, relapse rates following miltefosine treatment were higher among males of all ages and the sex difference was most pronounced after the age of 9. Furthermore, the authors could not identify gender-based differences in adherence to treatment protocol that would explain the sex-biased risk. Together, these results suggest an intrinsic male susceptibility to symptomatic VL [6].

Drug teratogenicity and decreased access to healthcare could potentially contribute to a lower rate of antileishmanial therapy among females in some endemic areas [61, 142]. In a study of PKDL patients in Bangladesh, miltefosine-induced corneal keratopathy was reported only in males. The authors suggest that increased treatment access could contribute to the male bias in this complication, although it cannot be excluded that biological differences might have played a role in the adverse effects observed among males [143].

A study from Kolkata, India, indicated no sex differences in the prevalence of VL but a significantly higher PKDL incidence in postpubertal males compared to females, as well as a more protracted course of PKDL among males. While this observation could be confounded by gender-related norms in health care use, studies of skin disease and wound healing in mice and humans have shown that estrogen promotes, whereas testosterone decreases skin healing [35, 36, 74]. There was also a significant positive correlation between levels of plasma testosterone and antileishmanial IgG in PKDL patients as measured by ELISA [74]. Increased levels of antileishmanial IgG have been implicated in the severity of PKDL [144]. Interestingly, after treatment with miltefosine, PKDL patients were found to have significantly lower plasma testosterone levels. A decrease in testosterone levels by miltefosine treatment may reduce the immunosuppressive effects of androgens, promoting an immune response more appropriate for parasite killing and disease resolution [13, 36, 74]. The authors concluded that these data highlight the importance of closely monitoring progression to PKDL in male VL patients, especially among pubertal and adult patients [74].

## 6. Concluding Remarks

The ultimate outcome of Leishmania infection will depend on a multitude of host-pathogen interactions and the development, or lack thereof, of immune responses that restrict parasite growth [2, 65]. Accumulating evidence shows sex-associated differences in the initiation and maintenance of immune responses [7, 13, 114, 118]. There are clearly social and epidemiological factors that contribute to differences in disease prevalence between the sexes in various endemic areas. Nonetheless, collective data from experimental models and studies of human infection suggest that there are also biological predispositions leading to sex-specific parasite burden and symptomatic disease during infection with *Leishmania* spp. Many epidemiological reports cannot easily differentiate between gender-related exposure and sex-based susceptibility. However, in Brazilian neighborhoods with peridomestic exposure, the data suggest that there are biological factors underlying the observed male bias in VL, as discussed herein [62]. In different endemic areas, whether a sex bias is present or not and whether males or females are more susceptible likely depend on the combination of the infecting species, epidemiological variables, and host biological factors. The molecular mechanisms underlying unique immune responses to infection are just beginning to be explored.

The increasing concern of drug-resistant pathogens, including but not limited to *Leishmania* spp., underscores the importance of developing therapeutic approaches that target host factors associated with disease progression. We propose that effective control and treatment of leishmaniasis and other infectious diseases should take into account the influence of biological sex in pathogenesis, immune response, and drug metabolism.

## Abbreviations

ADCL: Anergic diffuse cutaneous leishmaniasis
CCL: C-C motif chemokine ligand
CL: Cutaneous leishmaniasis
CR: Complement receptor
CYP: Cytochrome P450
DHT: Dihydrotestosterone
EREs: Estrogen-responsive elements
IFN-*γ*: Interferon-*γ*
IL: Interleukin
Ig: Immunoglobulin
LCL: Localized cutaneous leishmaniasis
LPG: Lipophosphoglycan
LPS: Lipopolysaccharide
*L.* (*V.*): *Leishmania* (*Viannia*)
MAPK: Mitogen-activated protein kinase
ML: Mucocutaneous leishmaniasis
MyD88: Myeloid differentiation primary response 88
NF*κ*B: Nuclear factor-*κ*B
NK cells: Natural killer cells
NO: Nitric oxide
PKDL: Post-kala-azar dermal leishmaniasis
R: Receptor
TGF-*β*: Transforming growth factor-*β*
Th: Helper T cell
TLRs: Toll-like receptors
TNF: Tumor necrosis factor
Treg: Regulatory T cell
VL: Visceral leishmaniasis
WT: Wild type.
